# High-frequency irreversible electroporation for cardiac ablation using an asymmetrical waveform

**DOI:** 10.1186/s12938-019-0693-7

**Published:** 2019-06-20

**Authors:** René van Es, Maurits K. Konings, Bastiaan C. Du Pré, Kars Neven, Harry van Wessel, Vincent J. H. M. van Driel, Albert H. Westra, Pieter A. F. Doevendans, Fred H. M. Wittkampf

**Affiliations:** 1 Div. Heart and Lungs, Dept. of Cardiology, University Medical Center Utrecht, Utrecht, The Netherlands; 2 Dept. of Medical Technology, University Medical Center Utrecht, Heidelberglaan 100, 3584 CX Utrecht, The Netherlands; 3 Witten/Herdecke University, Witten, Germany; 4 Dept. of Electrophysiology, Alfried Krupp Krankenhaus, Essen, Germany; 5 Dept. of Cardiology, Haga Teaching Hospital, The Hague, The Netherlands; 6 Abbott, Veenendaal, The Netherlands; 7 Holland Heart House, Utrecht, The Netherlands

## Abstract

**Background:**

Irreversible electroporation (IRE) using direct current (DC) is an effective method for the ablation of cardiac tissue. A major drawback of the use of DC-IRE, however, are two problems: requirement of general anesthesia due to severe muscle contractions and the formation of bubbles containing gaseous products from electrolysis. The use of high-frequency alternating current (HF-IRE) is expected to solve both problems, because HF-IRE produces little to no muscle spasms and does not cause electrolysis.

**Methods:**

In the present study, we introduce a novel asymmetric, high-frequency (aHF) waveform for HF-IRE and present the results of a first, small, animal study to test its efficacy.

**Results:**

The data of the experiments suggest that the aHF waveform creates significantly deeper lesions than a symmetric HF waveform of the same energy and frequency (*p* = 0.003).

**Conclusion:**

We therefore conclude that the use of the aHF enhances the feasibility of the HF-IRE method.

## Background

Radiofrequency (RF) energy is the clinically used thermal modality to perform ablations for the treatment of cardiac arrhythmias. Thermal ablation, however, has many limitations and can lead to severe complications such as pulmonary vein stenosis, phrenic nerve palsy or in rare cases to paraesophageal fistula [1]. Besides possible complications, the long-term efficacy of RF ablation as a therapy for atrial fibrillation is only about 65%. Irreversible electroporation (IRE) may be a safer and more effective method for cardiac ablation [2]. Over the past decade, preclinical studies have shown that IRE is a safe and valuable method for cardiac ablation therapy [3–11]. With IRE, a direct current (DC) is applied between a skin patch and a multipolar circular catheter. The high-intensity electric field near the catheter electrodes produces pores in the phospholipid membranes of the cells, leading to an irreversible breakdown of membrane structure and function and finally to cell death [12].

The direct current (DC), as used with IRE, leads to (skeletal) muscle contractions. When applying IRE in patients, the DC character of the applied current necessitates the use of general anesthesia. In addition, the DC current causes electrolysis on the electrode surfaces, thus producing bubbles containing the gaseous products from the electrolysis.

To prevent muscle stimulation, Arena et al. [13] studied the use of high-frequency (HF) alternating current for IRE and demonstrated the feasibility of HF-IRE for non-thermal ablation of brain tissue in rats without muscle contractions.

**Fig. 1:**
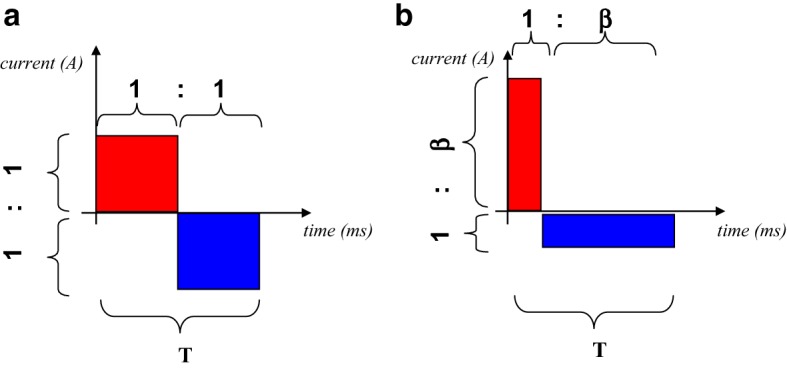
Schematics, showing a single wave period of a symmetric and an asymmetric waveform, having the same energy and the same time duration *T* of a single wave period. **a** Single wave period of a “symmetric waveform”. **b** Single wave period showing an adapted waveform in which the negative phase is flattened and spread out, while the positive phase is narrowed and higher (“asymmetric waveform”). The parameter *β* indicates the ratio of the duration of the long phase to the duration of the short phase

We have developed an *asymmetric* HF (aHF) waveform for IRE that is theoretically more effective than the (normal) symmetrical HF [14]. In this paper, our theoretical reasoning behind this asymmetrical waveform is explained, and in vivo experiments are presented that put this aHF waveform to the test. For clarity, throughout the paper, we will use the phrases “single wave period” and “wave train”, in which “single wave period” refers to a single short elementary wave period (having a duration of a few microseconds), whereas “wave train” refers to a series of many repeated contiguous single wave periods. These wave trains may themselves be repeated, so that the total treatment of a single patch of tissue may consist of a number of “wave trains”. The actual “asymmetry” of the aHF mentioned above takes place *within* each individual single wave period, as will be explained below using Fig. 1b.

The starting point for introducing our asymmetric waveform are remarks by, e.g., Son et al. indicating that electroporation is characterized by electrical creation of aqueous pores in cell membranes, in which the rate of the formation of these pores is strongly, and non-linearly, dependent on the transmembrane voltage [12, 15].

In our new method, we aim at exploiting this strongly non-linear, exponential, dependency on the transmembrane voltage, by applying an HF current pattern, in which, within each single wave period, one phase (e.g., the positive phase) has a much higher amplitude than the opposite phase. When compared to symmetrical HF, the high amplitude phase will then cause much more electroporation than the combined effect of both phases of symmetrical HF current, as explained below.

According to Krassowska et al. [12], the creation of electroporation pores in a membrane is proportional to $e^{{(\varPhi_{\text{m}} )^{2} }}$, in which *Φ*_m_ is the transmembrane voltage. We hypothesize that it is favorable for pore formation to have the highest phases of the applied electric field “work in the same direction”, as with a DC pulse, instead of having equal values in alternating directions as is the case with the symmetrical waveform (see Fig. 1a). The underlying fundamental reason for our hypothesis is the non-linear nature of pore formation, i.e., the fact that pore formation is proportional to $e^{{(\varPhi_{\text{m}} )^{2} }}$, which implies that, if the applied *Φ*_m_ would be increased with a factor 2, then the pore formation (and, consequently, cell death) would increase by a factor much larger than 2. Therefore, for enhancing the formation of electroporation pores, it is more effective to have a *higher* value of *Φ*_m_ during a *shorter* period of time, than a *lower* value of *Φ*_m_ that is spread out over a *longer* period of time.

Since the total charge released from the electrode during one single wave period (consisting of one positive and one negative phase) must always be exactly equal to zero (to avoid unwanted DC current), we introduce an asymmetric waveform (see Fig. 1b) in which, for each single wave period, the surface area of the positive phase is equal to the surface area of the negative phase. Given the above-mentioned fact that the creation of new electroporation pores in a membrane is proportional to $e^{{(\varPhi_{\text{m}} )^{2} }}$, the positive phases of the asymmetrical wave, shown in Fig. 1b, are much more effective in pore creation compared to those of the symmetrical wave, shown in Fig. 1a, and hence the total net effect of the entire asymmetric wave is expected to work in one single direction (i.e., the direction of the positive phase), not unlike a DC pulse in that direction.

Therefore, *our first hypothesis* reads that the asymmetrical waveform (Fig. 1b) is more effective for IRE than the symmetrical waveform (Fig. 1a) of the *same energy and frequency*.

To set up a meaningful comparison of both waveforms, it is obvious to demand that the asymmetric and symmetric waveforms have the same energy within a single period of the wave. However, another obvious choice is to demand that, for both waveforms, each single (positive or negative) phase represents the same amount of charge (positive or negative) released from the electrodes, because, for frequencies well above 10 kHz, this amount of charge determines whether unwanted muscle spasms are evoked [13].

Therefore, *our second hypothesis* reads that the asymmetrical waveform (Fig. 1b) is more effective for irreversible electroporation than a symmetrical waveform of the same frequency if the positive (or negative) phase of the asymmetric waveform *has the same charge* as the positive (or negative) phase of the symmetric waveform. The charge of a positive phase of a waveform is represented by the area under the curve during that positive phase.

Furthermore, there are at least two boundary conditions that need to be met when designing an asymmetric waveform:

The *first boundary condition* is that the duration of one single period must be much shorter than 100 µs (the chronaxie time of fast neurons [16]), to be able to avoid muscle spasms using the reasoning presented above.

The second boundary condition relates to the mechanism of building transmembrane voltages:

As explained by, e.g., Golberg et al. [17], the currently accepted theory is that, at the very beginning of each new (positive or negative) phase of a waveform representing the current injected from the electrode, a process starts that consists of at least two consecutive phases: (i) a brief initial phase during which the applied current causes the accumulation of charge (and hence the buildup of the transmembrane voltage *Φ*_m_) on the membranes of cells, and (ii) the actual formation of pores in the membranes. Son et al. [18] present a simulation using an applied field strength of 1.5 kV/cm near the membrane, in which the first phase (i) has a duration of 0.7 μs, after which a transmembrane voltage of 1.2 V is reached and a burst of pore formation ensues.

Therefore, the *second boundary condition* reads that duration of the shortest phase in the asymmetric wave must be much larger than the time needed to build up the transmembrane voltage of 1.2 V, in which the numbers used in the study by Son et al. serve as a guideline.

On the basis of mathematical analysis alone, it can be shown that if the symmetrical and the asymmetrical wave contain equal energy, then the *symmetrical wave has more charge* than the asymmetrical wave during a *single phase of one single period* of the wave. See Appendix for the mathematical derivation.

We therefore conclude that if *our first hypothesis* is confirmed by experiment, then *our second hypothesis* is confirmed automatically as well.

In the present study, the efficacy of both waveforms is analyzed on the basis of the size of the lesions that they produce.

## Methods

### High-frequency waveforms

The symmetric and asymmetric waveforms have been designed to comply with the two boundary conditions mentioned in the introduction, by choosing a frequency of 167 kHz.

The waveforms were generated using an HF pulse generator (HF-Puls 4, GBS Elektronik, Großerkmannsdorf, Germany). The generator was altered to enable the generation of asymmetrical pulses. The fact that we used an existing HF pulse generator as a starting point for our alterations, however, posed a limitation to the degree of asymmetry that could be achieved: the ratio of the duration of the long phase to the duration of the short phase, within a single wave period, could not exceed 2.6:1.

The asymmetric waveforms were composed in such a way that the asymmetric and symmetric waveforms have the same energy within a single period of the wave. In this way, the experiments are designed to test for the *first hypothesis* mentioned in “Background”.

Two different types of experiments have been performed: (i) the main group of experiments, using ten consecutive wave trains (performed on animal groups M1 and M2, see Table 1), and an additional experiment (ii) using only one single wave train of high energy (performed on animal group E).

**Table 1:** HF pulse protocol

	5 animals (model M)	
	Symmetrical	Asymmetrical (*β* = 2.31)
Positive voltage peak (V)	600	544
Negative voltage peak (V)	− 600	− 221
Frequency (kHz)	167	167
Number of wave trains	10	10
Interval (ms) between wave trains	400	400
Duration (ms) of single wave train	2	2
Delivered energy	10 × 20 J	10 × 20 J
Energy during one single wave period *T*	100%	100%
Charge during one single wave period *T*	100%	92%

### Animals

All animal experiments were performed with prior approval from the Animal Experimental Ethical Committee of the University Medical Center Utrecht (protocol number AVD115002015206).

**Fig. 2:**
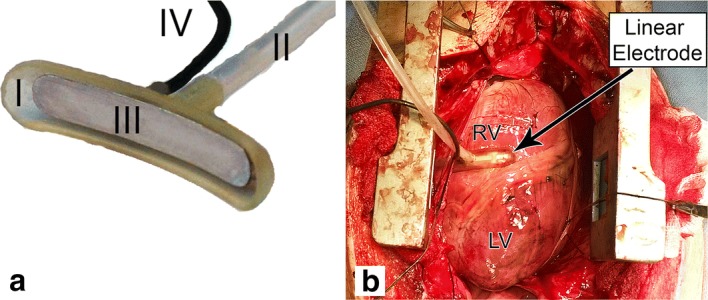
**a** The used suction cup electrode showing the 42 × 7 mm suction cup (I) connected to a vacuum system (II), containing a 35 × 6 mm stainless steel electrode (III) that is connected to the HF generator by a cable (IV). **b** Placement of the suction cup electrode on the right ventricle. *LV* left ventricle, *RV* right ventricle

In all models, 60–75 kg Dalland landrace pigs were premedicated with 10 mg/kg ketamine, 0.4 mg/kg midazolam and 0.5 mg atropine. Anesthesia was induced with 4 mg/kg thiopental sodium. During the rest of the procedure, 0.5 mg/kg/h midazolam, 2.5 mg/kg/h sufentanil and pancuronium bromide 0.1 mg/kg/h were administered. A midline sternotomy was performed and the pericardial sac was opened. Subsequently, the suction cup electrode (Fig. 2) was positioned on the right ventricle (RV) perpendicular to the left anterior descending coronary artery because of the visibility and ease of access. Then either one of the HF-IRE waveforms was applied (Table 1), and sutures were placed on both ends of the electrode. Successively, the suction cup electrode was repositioned 1 cm toward the apex and the other HF-IRE waveform was applied, and again sutures were placed.

### Five animals for the main experiment (M)

#### Animal model M1

In two animals, 8 h after HF-IRE energy application, the animals were exsanguinated and the heart was removed for histological analysis.

#### Animal model M2

Three animals were pretreated with amiodarone, 800 mg/day, for 7 days before the procedure. After the administration of the HF-IRE, the pericardium and thorax were closed. Three weeks later the animals were exsanguinated and the heart was removed for histological analysis.

### Additional animals for high-energy experiment (E)

In two additional animals, we repeated the experiments described above (experiment M), but all currents in e experiment (E) were two times stronger than the corresponding currents in the experiment (M), and, furthermore, the number of wave trains (10 wave trains in experiment (M), see Table 1), was reduced to just one single wave train. Besides these two changes in HF application, the experimental setup and follow-up time were the same as in model M2.

### Histological analysis

In both groups, RV tissue containing the ablated area was isolated from the rest of the heart and fixated in a 4% formaldehyde solution for at least 3 days. From each lesion, two histological samples were taken at 1/3 and 2/3 along the length of the ablation site. Histological sections were prepared and stained with both hematoxylin and eosin (HE) and Elastic van Gieson (EvG). Lesion depth and RV wall thickness were scored by a blinded observer as described previously [8].

### Statistical analysis

The impedance was calculated as the set peak-to-peak voltage divided by the measured peak-to-peak current. For the histology, the mean of the four measurements per animal per lesion was used for further analysis. A paired two tailed *t* test was used to compare lesion depth for both methods. A *p* value < 0.05 was considered to be statistically significant.

## Results

The HF-IRE applications were successfully delivered in all experiments without medical complications. Minor skeletal muscle contractions were observed with all HF-IRE applications. Visual inspection of the ablation sites directly after IRE application always showed a hyperemic mark left by the suction cup. There were no signs of tissue heating (e.g., tissue whitening).

**Fig. 3:**
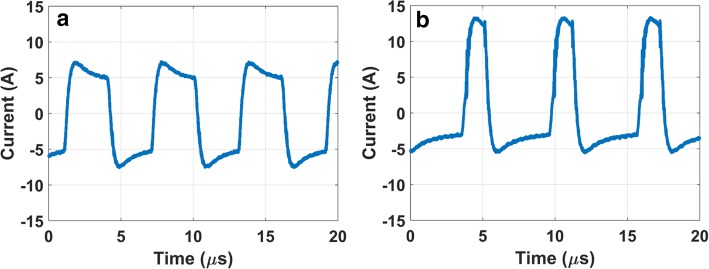
Current strength (in amperes) produced in the experiments of model M, as function of time in microseconds. **a** Symmetrical waveform. **b** Asymmetrical waveform

In Fig. 3, the current delivered via the electrodes during the experiments is rendered as a function of time, for the symmetric wave (left) and the asymmetric wave (right). The calculated impedance was not significantly different at 107 ± 18 Ω and 97 ± 20 Ω (*p* = 0.137) for the symmetric HF (sHF) and asymmetric HF (aHF) waveforms, respectively.

### Histological analysis

In one animal of model E, due to adhesions, the epicardium was damaged during excision of the heart. This animal was excluded from further histological analysis. A total of 28 histological slides from seven animals were analyzed. There was no significant difference in the thickness of the RV myocardium at the lesion sites, 7.7 ± 2.2 mm and 5.9 ± 1.5 mm (*p* = 0.15) for the sHF and aHF sites, respectively.

### Model M

**Fig. 4:**
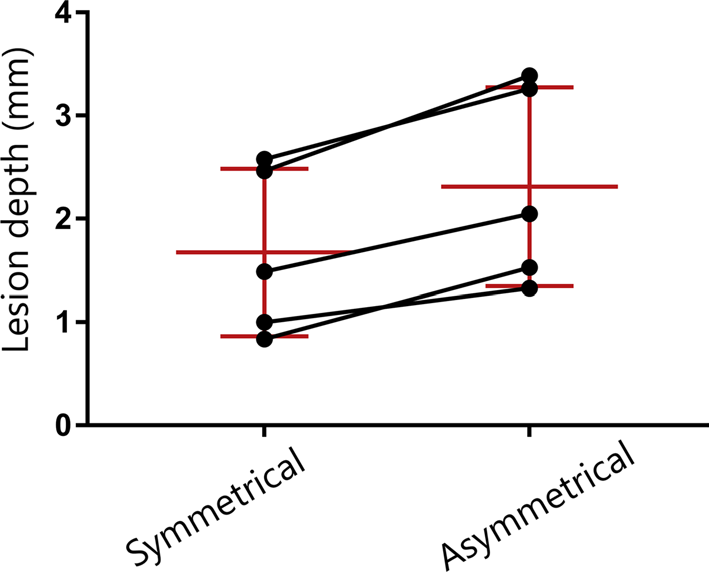
Lesion depth of the symmetrical and asymmetrical HF ablations in model M (*n* = 5). The black lines indicate the paired measurements; red lines indicate the mean and standard deviation per group

In model M1, 8 h after HF-IRE, the sHF and aHF waveforms showed a lesion depth of 1.2 ± 0.3 mm and 1.8 ± 0.3 mm (*p* = 0.068), respectively. In model M2, 3 weeks after HF-IRE, the sHF and aHF showed a lesion depth of 2.0 ± 0.9 mm and 2.7 ± 1.2 mm (*p* = 0.065), respectively. See also Table 2. Overall, for all five animals of Model M, the asymmetrical waveform shows a significantly deeper average lesion depth of 2.3 ± 0.9 mm compared to the symmetrical waveform 1.7 ± 0.7 mm (*p* = 0.003), Fig. 4. The overall average relative lesion depth was significantly deeper for the asymmetrical waveform aHF lesions (43.1 ± 19.4%) than for the symmetrical waveform sHF lesions (21.4 ± 7.2%) (*p* < 0.05). With the sHF waveform, there were no transmural lesions (range: 11.6–31.6%). With the aHF waveform, there was one transmural lesion (range: 13.2–100%).

**Table 2:** Results

	Lesion depth (mm)		*p*-value
	Asymmetrical HF	Symmetrical HF	
Model M1 (*n* = 2)	1.8 ± 0.4	1.2 ± 0.5	0.068
Model M2 (*n* = 3)	2.7 ± 1.2	2.0 ± 0.9	0.065
Overall M (*n* = 5)	2.3 ± 1.0	1.7 ± 0.8	0.003

### Model E

The average RV myocardial thickness was 10.0 ± 1.1 mm and 8.4 ± 4.6 mm (*p* = 0.634) in the sHF and aHF groups, respectively. In model E, 3 weeks after HF-IRE, the sHF and aHF showed a lesion depth 3.9 ± 0.4 mm and of 3.8 ± 1.5 mm (*p* = 0.911), respectively. The average relative lesion depths were 38.1 ± 2.0% and 55.0 ± 9.3% (*p* = 0.281) for the sHF and aHF lesions, respectively.

## Discussion

In the present porcine study, we demonstrated the feasibility of a novel asymmetrical aHF waveform for IRE, and compared it directly to a conventional, symmetrical sHF waveform. This is the first time that an asymmetrical wave has been utilized for IRE ablation and has shown to be usable for non-thermal ablation of cardiac tissue. In the animals that received multiple low-energy HF-IRE application, the aHF waveform led to significantly deeper lesions than the sHF waveform. As a result, we conclude that the *first hypothesis* from “Background” has been confirmed, i.e., an asymmetrical high-frequency waveform creates deeper lesions than a symmetrical waveform of the same energy. In Appendix, we show that if the *first hypothesis* is confirmed, then the *second hypothesis* (i.e., an asymmetric high-frequency waveform creates deeper lesions than a symmetric waveform having the same charge per phase) is confirmed automatically as well.

Our findings are in accordance with the strength–duration relation for the irreversible lesion of cardiomyocytes by high-intensity electric fields as presented by Prado et al. [15], in which the strength of the electric field necessary to kill 50% of the cells, *E*_50_, was fitted to their measured data using *E*_50_ = *Kd*^*A* + *B*, in which *d* is the duration of the applied field, and *A* = − 0.54, thus indicating a strongly non-linear relation between *E*_50_ and *d*.

In the two animals that received one single high-energy HF application, there was no significant difference between aHF and conventional HF, but both methods showed lesions of more than 4 mm deep. With all HF-IRE applications, we observed minor skeletal muscle contractions; these were however considerably less forceful than those observed with DC-IRE applications. A bench test of the HF generator revealed a small unintended low-frequency component (< 500 Hz) in the waveform. This low-frequency wave is assumed to be responsible for the observed minor muscle contractions in the present in vivo experiments.

For the purpose of pulmonary vein isolation, lesions of a few millimeters deep are required to ensure a transmural ablation of the usually thin myocardial sleeves. The use of a single high-energy aHF-IRE application [high-energy experiment (E)] has been shown to be capable of producing lesions that are deep enough for pulmonary vein isolation. However, these lesions were created by applying the HF current between a skin patch and an electrode in a suction cup. When applying this HF current between the skin patch and a circular multi-electrode catheter, due to the low resistance of blood, about 2/3 of the energy dissipates into the blood pool instead of the tissue. Therefore, when using a circular multi-electrode catheter instead of a suction cup, considerably higher currents will be required.

In this study, the aHF and sHF applications provoked extremely minor skeletal muscle contractions, especially compared to DC-IRE applications observed in previous studies [1–6]. For high frequencies, i.e., frequencies that are well above 1/Tchron, in which Tchron is the chronaxie time, the neurostimulation threshold is essentially proportional to the frequency. Therefore, high frequencies, as used in the present study, are less likely to stimulate skeletal muscles.

To investigate whether or not bubble formation (due to electrolysis) takes place using our symmetric or asymmetric high-frequency protocols, we performed extensive testing in a saline bath and studied the surfaces of the electrodes during the pulses. No visible bubble formation on or near the electrode surfaces was found.

### Limitations

The number of animals used for this pilot study was small; however, significant differences between the two study groups were obtained. We did not quantitatively assess skeletal muscle contraction, instead assessment was performed visually. Future studies on HF-IRE should incorporate measurements of peak acceleration to quantitatively assess skeletal muscle contractions.

Higher energies are required when using this HF technology for clinical ablation procedures. In the present study, we were unable to test higher energy settings due to limitation of the experimental generator.

### Clinical perspective

The depth of the lesions created with the asymmetric HF waveform in these pilot experiments may not yet be sufficient for the creation of transmural scars in the ostia of the pulmonary veins. We expect that using a higher power and a higher value for β in the asymmetric pulse will create lesions suitable for pulmonary vein isolation. Besides the application of aHF-IRE for pulmonary vein isolation, other applications in the field of cardiac electrophysiology are possible, for example with epicardial approaches [7].

We expect that a substantially higher value for β in the asymmetric pulse will boost the efficacy of the aHF-IRE even further, thus making it a suitable alternative for DC-IRE, because aHF-IRE will avoid skeletal muscle contractions and gas bubble formation due to electrolysis; further studies are however required to prove this.

Irreversible electroporation using various waveforms is increasingly being investigated for the purpose of cardiac ablation. The first clinical trials have been performed using (pulsed) high-voltage waveform that used general anesthesia to avoid skeletal muscle stimulation [19]. Besides the present study, other research into symmetrical biphasic waveforms is being conducted [20]. In contrast to the present study, both approaches apply the pulses between multiple catheter electrodes, obviating the need for a skin patch. While the approach could lead to less skeletal muscle stimulation, it may have ramifications for the size of the created lesions considering the resulting shape of the electric field.

## Conclusion

The present study demonstrates that the novel asymmetric high-frequency waveform can create lesions in cardiac tissue. The data of the experiments also suggest that an asymmetric high-frequency waveform creates deeper lesions than a symmetric waveform of the same energy, or a symmetric waveform of the same charge.

## Figures and Tables

**Fig. 5:**
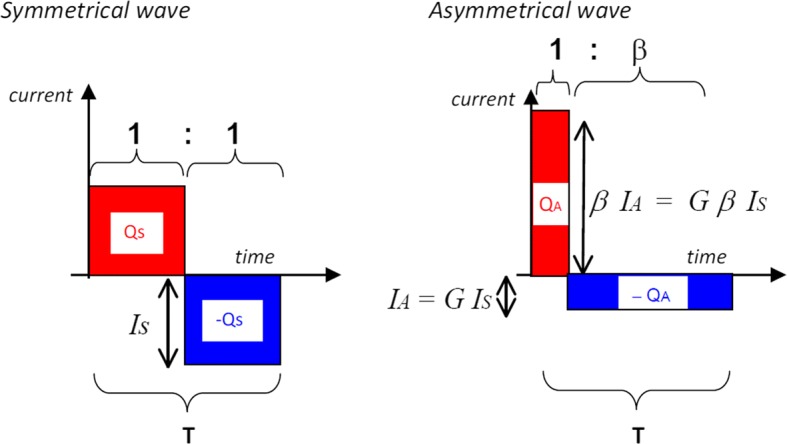
Graphical rendering of the role of the parameters *β* and *G* in the asymmetrical waveform

**Fig. 6:**
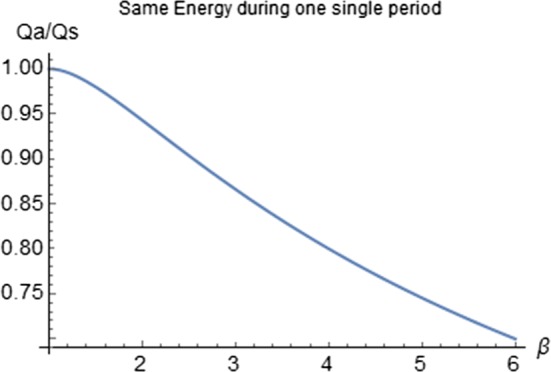
Plot of *Q*_a_/*Q*_s_ as function of *β.* For all values of *β* > 1, the ratio *Q*_a_/*Q*_s_ < 1, which implies that Qa will always be smaller than *Q*_s_ if *β* ≠ 1

## Data Availability

Not applicable.

## Appendix

In this appendix, we show that, for all values of *β* > 1, the following statement is true: if the symmetrical and the asymmetrical wave contain equal energy, then the *symmetrical wave has more charge* than the asymmetrical wave during a *single phase of one single period* of the wave.

To prove the validity of this statement, we first calculate the amplitudes needed for the asymmetrical wave to let its energy be equal to the energy of the symmetrical wave.

### Equal energy

Let *I*_S_ denote the amplitude of the symmetrical wave. The energy *E*_S_ that is released during a single period of the symmetrical wave then reads:

1 $$ E_{\text{S}} = \left( {I_{\text{S}} } \right)^{2} RT, $$

in which *R* is the resistance between the electrodes and *T* is the duration of a single period of the wave.

Let *I*_A_ denote the amplitude of the longest period of the asymmetrical wave (see Fig. 5), which can be tuned using a controllable gain factor *G*:

2 $$ I_{\text{A}} = GI_{\text{S}} . $$

The longest phase of a single period of the asymmetrical wave has amplitude *I*_A_ and duration *Tβ*/(1 + *β*), and hence releases a charge equal to *I*_A_(*Tβ*)/(1 + *β*) and an energy equal to

3 $$ \left( {I_{\text{A}} } \right)^{2} {{RT\beta } \mathord{\left/ {\vphantom {{RT\beta } {\left( {1 + \beta } \right)}}} \right. \kern-0pt} {\left( {1 + \beta } \right)}}. $$

However, the shortest phase of a single period of the asymmetrical wave has amplitude *βI*_A_ and duration *T*/(1 + *β*), and hence releases a charge—(*βI*_A_)*T*/(1 + *β*), which is equal to the charge of the longest phase but with opposite sign, and releases an energy equal to

4 $$ \left( {\beta I_{\text{A}} } \right)^{2} {{RT} \mathord{\left/ {\vphantom {{RT} {\left( {1 + \beta } \right)}}} \right. \kern-0pt} {\left( {1 + \beta } \right)}}. $$

In this way, the total charge released during the entire single period of the asymmetrical wave equals zero, which is a prerequisite for avoiding unwanted low-frequency effects such as neurostimulation and electrolysis.

The total energy *E*_A_ released during the entire single period of the asymmetrical wave now equals

5 $$ E_{\text{A}} = \left( {I_{\text{A}} } \right)^{2} RT\beta , $$

as can be seen by adding the expressions (3) and (4) above.

### Gain factor for asymmetrical wave to enforce equal energy for symmetrical and asymmetrical wave

As has been indicated above, a controllable gain factor *G* for the asymmetrical wave can now be used to ensure that *E*_A_ = *E*_S_, which corresponds to the *first hypothesis* mentioned in “Background”.

As can be seen from combining Eqs. (2) and (5), the demand *E*_A_ = *E*_S_ entails that the gain factor *G* must be tuned to

6 $$ G = {1 \mathord{\left/ {\vphantom {1 {\surd \beta }}} \right. \kern-0pt} {\surd \beta }}. $$

Now that it is ensured that *E*_A_ = *E*_S_, we can now evaluate the charge *Q*_A_ that is released during a *single phase* out of a single period of the *asymmetrical* wave and compare this charge *Q*_A_ to the charge *Q*_S_ released during a *single phase* out of a single period of the *symmetrical* wave.

Substituting (6) into (2) yields *I*_A_ = *I*_S_/√*β*, and hence

7 $$ Q_{\text{A}} = {{I_{\text{A}} \left( {T\beta } \right)} \mathord{\left/ {\vphantom {{I_{\text{A}} \left( {T\beta } \right)} {\left( {1 + \beta } \right)}}} \right. \kern-0pt} {\left( {1 + \beta } \right)}} = {{\left( {{{I_{\text{S}} } \mathord{\left/ {\vphantom {{I_{\text{S}} } {\surd \beta }}} \right. \kern-0pt} {\surd \beta }}} \right)\left( {T\beta } \right)} \mathord{\left/ {\vphantom {{\left( {{{I_{\text{S}} } \mathord{\left/ {\vphantom {{I_{\text{S}} } {\surd \beta }}} \right. \kern-0pt} {\surd \beta }}} \right)\left( {T\beta } \right)} {\left( {1 + \beta } \right)}}} \right. \kern-0pt} {\left( {1 + \beta } \right)}}. $$

Since *Q*_S_ simply equals *Q*_S_ = *I*_S_*T*/2, we now have our main result:

8 $$ {{Q_{\text{A}} } \mathord{\left/ {\vphantom {{Q_{\text{A}} } {Q_{\text{S}} }}} \right. \kern-0pt} {Q_{\text{S}} }} = {{\left( {{{\left( {{{I_{\text{S}} } \mathord{\left/ {\vphantom {{I_{\text{S}} } {\surd \beta }}} \right. \kern-0pt} {\surd \beta }}} \right)\left( {T\beta } \right)} \mathord{\left/ {\vphantom {{\left( {{{I_{\text{S}} } \mathord{\left/ {\vphantom {{I_{\text{S}} } {\surd \beta }}} \right. \kern-0pt} {\surd \beta }}} \right)\left( {T\beta } \right)} {\left( {1 + \beta } \right)}}} \right. \kern-0pt} {\left( {1 + \beta } \right)}}} \right)} \mathord{\left/ {\vphantom {{\left( {{{\left( {{{I_{\text{S}} } \mathord{\left/ {\vphantom {{I_{\text{S}} } {\surd \beta }}} \right. \kern-0pt} {\surd \beta }}} \right)\left( {T\beta } \right)} \mathord{\left/ {\vphantom {{\left( {{{I_{\text{S}} } \mathord{\left/ {\vphantom {{I_{\text{S}} } {\surd \beta }}} \right. \kern-0pt} {\surd \beta }}} \right)\left( {T\beta } \right)} {\left( {1 + \beta } \right)}}} \right. \kern-0pt} {\left( {1 + \beta } \right)}}} \right)} {\left( {I_{\text{S}} T/2} \right)}}} \right. \kern-0pt} {\left( {I_{\text{S}} T/2} \right)}} = {{2\surd \beta } \mathord{\left/ {\vphantom {{2\surd \beta } {\left( {1 + \beta } \right)}}} \right. \kern-0pt} {\left( {1 + \beta } \right)}}. $$

As can be seen from the plot of 2√*β*/(1 + *β*) as a function of *β* > 1 (see Fig. 6), we have that

9 $$ {{Q_{\text{A}} } \mathord{\left/ {\vphantom {{Q_{\text{A}} } {Q_{\text{S}} }}} \right. \kern-0pt} {Q_{\text{S}} }} = {{2\surd \beta } \mathord{\left/ {\vphantom {{2\surd \beta } {\left( {1 + \beta } \right)}}} \right. \kern-0pt} {\left( {1 + \beta } \right)}} < 1\quad {\text{for}}\;{\text{all}}\;{\text{values}}\;{\text{of}}\;\beta > 1. $$

Therefore, we now reach our main conclusion that:

10 $$ {\text{If}}\;E_{\text{A}} = E_{\text{S}} \;{\text{then}}\;Q_{\text{A}} < Q_{\text{S}} \quad {\text{for}}\;{\text{all}}\;\beta > 1. $$

This conclusion (10) implies that, if the *first hypothesis*, mentioned in “Background”, is fulfilled, i.e., if the lesion using the asymmetrical wave is deeper than the lesion using the symmetrical wave under the condition *E*_A_ = *E*_S_, then even more energy for the asymmetrical wave, i.e., *E*_A_ > *E*_S_, would be needed to satisfy the condition *Q*_A_ = *Q*_S_ for the *second hypothesis*, which makes the lesion from the asymmetrical wave even deeper than the lesion of the first hypothesis.

Therefore we conclude that:

If the *first hypothesis* is fulfilled, then the *second hypothesis* is fulfilled automatically as well.
